# The neuroscience of body memory: Recent findings and conceptual advances

**DOI:** 10.17179/excli2023-5877

**Published:** 2023-02-07

**Authors:** Claudia Repetto, Giuseppe Riva

**Affiliations:** 1 Department of Psychology, Università Cattolica del Sacro Cuore, Milan, Italy; 2 Humane Technology Lab, Università Cattolica del Sacro Cuore, Milan, Italy; 3 Applied Technology for Neuropsychology Lab, Istituto Auxologico Italiano IRCCS, Milan, Italy

**Keywords:** body representation, body memory, body matrix, advanced technologies, interoception, bodily illusions

## Abstract

The body is a very special object, as it corresponds to the physical component of the self and it is the medium through which we interact with the world. Our body awareness includes the mental representation of the body that happens to be our own, and traditionally has been defined in terms of body schema and body image. Starting from the distinction between these two types of representations, the present paper tries to reconcile the literature around body representations under the common framework of body memory. The body memory develops ontogenetically from birth and across all the life span and is directly linked to the development of the self. Therefore, our sense of self and identity is fundamentally based on multisensory knowledge accumulated in body memory, so that the sensations collected by our body, stored as implicit memory, can unfold in the future, under suitable circumstances. Indeed, these sets of bodily information had been proposed as possible key factors underpinning several mental health illnesses. Following this perspective, the Embodied Medicine approach put forward the use of advanced technologies to alter the dysfunctional body memory to enhance people's well-being. In the last sections, recent experimental pieces of evidence will be illustrated that targeted specifically bodily information for increasing health and wellbeing, by means of two strategies: interoceptive feedback and bodily illusions.

See also Figure 1(Fig. 1).

## Introduction

We all have a body, which is the physical component of the self. Empirical data shows that the mind and body are strongly connected and influence each other: on the one hand, the body, in its perceptual and motor parts, affects cognition (Wilson, 2002[91]; Barsalou, 2008[4]); on the other hand, bodily states, which are the building blocks of the conscious experience, are linked to the development of the self (Riva 2018[69]). Cognitive and neuroimaging studies show that the body is more important than any other object in the world. For example, behavioral evidence showed that there are specific mechanisms, systems, and resources for encoding and remembering visually perceived body-related stimuli (Galvez-Pol et al., 2020[33]). Also, our body is the point of reference for how we experience events and how we remember them later. Events experienced from inside the body (first-person perspective) are more likely to be remembered from the same perspective, while events experienced from outside the body (third-person perspective) are more likely to be remembered from the same perspective (Bergouignan et al., 2022[5]).

The purpose of this paper is to update knowledge about body memory and possible ways to change it to improve well-being and health. First, we will define body representations, then illustrate a recent model that describes the possible relationships between their various components and provide empirical evidence to support that model. The body self-consciousness framework will be introduced in the second section, which advances the connection between the body and the self. We will connect this approach with the concept of the body matrix and its neural underpinnings after briefly summarizing the ontogenic development of body memory. Finally, we will discuss two strategies for altering the body matrix with advanced technologies to improve mental health: interoceptive feedback and bodily illusions.

## From Body Representations to Body Memory

Our body is the medium through which we interact with the world. For this interaction to be effective, our body must accomplish at least two main tasks: i.) to collect information from the environment (through the afferent sensory systems); ii.) to make actions within the environment (through the efferent motor system). However, these tasks could not be performed if we did not have a sense of our body (Gallese and Sinigaglia, 2010[32]), which is a mental representation of the body that happens to be our own (Gallagher, 2006[31]). This body awareness has been described in terms of body representations (de Vignemont, 2010[19]; Gadsby, 2019[27]; Sorrentino et al., 2021[87]). Traditionally, two distinct body representations have been identified, the *body schema* and the *body image *(Gallagher, 1986[30]; Dijkerman and de Haan, 2007[22]; de Vignemont, 2010[19]). The body schema was originally defined by Head as a body posture model organizing "the impressions produced by incoming sensory impulses in such a way that the final sensation of position, or of locality, rises into consciousness charged with a relation to something that has happened before" [Head, 1920[37], cited in Gallagher (1986[30])]. In this view, movement-directing sensorimotor representations of the body (e.g. position, size, strength) make up the body schema, whose main function is underpinning actions. On the other hand, the body image includes the representations related to perception (e.g. structure and shape of the body). 

This clearcut distinction, though has received considerable acclaim in the literature, posits some challenges at both the neural and theoretical levels. First, the functional distinction between these two different body representations perfectly matched with the action-perception dichotomy of the somatosensory systems, originally proposed for the visual system (Milner and Goodale, 1995[52]), and postulating the segregation of the information in two pathways, one for action planning and one for somatosensory perception and recognition (Dijkerman and de Haan, 2007[22]). According to this model, the stream concerned with action-related processing reaches the posterior parietal cortex, whereas the stream dedicated to perception and recognition projects from the anterior parietal cortex to the posterior insula through the secondary somatosensory cortex. However, empirical evidence demonstrated that this model is too restrictive: specifically, the independence of processing between the two streams, the limitation to only two paths, and the tenet of information encapsulation have been questioned (Ehrsson et al., 2004[24]; Schenk and McIntosh, 2010[78]; Gallace and Spence, 2010[29]). More recently, de Haan and Dijkerman (2020[22]) put forward a novel theory of the functional organization of somatosensory processing that includes a number of specialized routes far more multimodal in nature and much less independent from each other. This model comprises a 'cylinder block' of basic somatosensory processing and five higher-order networks involved in: (i) haptic object recognition and memory; (ii) body perception; (iii) body ownership; (iv) affective processing; and (v) action. 

In addition, from a theoretical perspective, some scholars have highlighted the need to question the links between the two types of body representations (Pitron and de Vignemont, 2017[62]; Pitron et al., 2018[61]), in particular asking whether they are independent or co-constructed, and to what extent they communicate with each other. To answer these questions, Pitron and DeVignemont (2017[62]) first underlined an important distinction between short-term body representations (e.g. posture), built in the online experience with the world, and long-term body representations (e.g. bodily configuration and metrics). The latter includes an implicit model of the body properties stored in the long-term memory (Longo and Haggard, 2012[47]), which is updated across the lifespan, integrating multisensory signals coming from the sensorimotor and proprioceptive systems. Indeed, a study directly comparing the body representations in young adults and the elderly with both implicit (body-landmark localization task) and explicit (avatar adjustment task) tasks revealed that only older individuals' implicit and explicit upper limbs body representations were distorted (Sorrentino et al., 2021[87]). According to the authors, this effect could be due to an age-related decline in the amount and richness of sensory inputs and motor function, making bodily information less efficiently processed. 

Focusing on long-term body representations, Pitron and colleagues (Pitron and de Vignemont, 2017[62]; Pitron et al., 2018[61]) compared three theoretical models dealing with mainly two broad issues: i. does the distinction between body image and body schema hold for long-term body representations? and if so, ii. how do they interact with each other? On one side there is the fusion model, proposing a multifunction unique representation that merges action features and perceptual properties of the body (O'Shaughnessy, 2002[56]; Gadsby, 2017[28]). The opposite view is put forward by the independence model, which claims the existence of two separate representations for action and perception, and postulates they work independently from each other. An intermediate position is suggested by the co-construction model (Pitron and de Vignemont, 2017[62]; Pitron et al., 2018[61]), according to which body image and body schema are inherently distinct within long-term body representations, but they are intertwined as they reshape each other using iterative loops of Bayesian computations. An increasing number of experimental studies provided support to this model (Irvine et al., 2019[40]; Caggiano and Cocchini, 2020[11]; Raimo et al., 2021[66][67]). Irvine and co-workers (2019[40]), trying to elucidate how body image and body schema interact in a healthy population, found that body image mediates the performance on a motor imagery affordance task, and the body schema was influenced by body image, with a specific moderator role of the personal attitudes toward the body. Similarly, extending the investigation to the whole body, Caggiano and Cocchini (2020[11]) identified a systematic difference in the representation of the lower versus upper body parts, being the lower limbs overestimated and the upper limbs underestimated. These findings have been interpreted as the outcome of the interplay between body parts and their functional role: namely, the body image of a specific body part is influenced by its function during action execution (body schema). Raimo and colleagues (2021[66][67]) studied body representations in relation to interoceptive sensitivity in different age groups and reported worse performance in both body schema and body image tasks for children and the elderly compared to young adults, with a specific mediator effect of interoceptive sensitivity only on the relation between age and body schema. These findings seem consistent with the co-construction model in that they support the idea that the two body representations reshape each other, and the body schema is more influenced than the body image by interoceptive signals. 

The interest in long-term body representations and their malleability to accommodate new experiences has grown in different disciplines, from cognitive psychology to neuropsychology, neuroscience, and philosophy. Recently Riva (2018[69]) has drawn attention to the concept of *body memory* as a common label framing diverse perspectives in the study of body representations. One of the first scholars to introduce the term body memory was Prof. Thomas Fuchs whose aim was to combine a philosophical approach based on phenomenology with psychiatry. In its seminal work (Fuchs, 2012[26], p. 9), the author underlines that “*Memory comprises not only one's explicit recollections of the past, but also the acquired dispositions, skills, and habits that implicitly influence one's present experience and behavior. This implicit memory is based on the habitual structure of the lived body, which connects us to the world through its operative intentionality*”. In his view, body memory is distinguished into different components, namely procedural, situational, intercorporeal, incorporative, pain, and traumatic memory. Our sense of self and identity is fundamentally based on the structures accumulated in body memory, and the sensations collected by our body, stored as implicit memory, can unfold in the future, under suitable circumstances. Indeed, these sets of bodily information had been proposed as possible key factors underpinning several mental health illnesses (Koch et al., 2013[45]). Following this line of reasoning, recently Gentsch and Kuehn (2022[35]) examined the hypothesis that negative body memories, in the form of adverse bodily experiences stored in the implicit memory, give rise to Clinical Body Memory mechanisms, such as trauma, pain, dissociation, and other somatic symptoms. 

The focus on multisensory body experiences as the grounding of body memory is shared also with the fields of cognitive psychology and clinical neuroscience (Gaudio and Quattrocchi, 2012[34]; de Haan and Dijkerman, 2020[18]). However, in Riva's view (2018[69], p. 253), the concept of body memory “directly connects the experience of the body with the intentional development of the self”, therefore it can be considered a bridge between the above-described cognitive approach (which is concerned with the bodily experiences) and the volitional approach (focused on the sense of self and its intentionality). In the next section, the role of body memory in self-construction will be discussed, with a specific focus on how to use technologies to alter dysfunctional body memory.

## From the Body to the Self

When we experience the world through our bodies we feel like the subjects of a conscious experience, that is the impression that one's experiences as a sentient being are connected to the self and comprise one single entity (the “I”) (Ronchi et al., 2018[75]). This bodily self, based on multisensory brain mechanisms integrating both exteroceptive and interoceptive body signals, has been defined as body self-consciousness (BSC) (Blanke et al., 2015[7]). Blanke and colleagues (2015[7]) reviewing literature data coming from human behavior, animal neurophysiology, human neuroimaging, and computational models, proposed the existence of two distributed and partially overlapping cortical networks of BSC: one more anterior, located in the frontoparietal cortex, processing signals related to body-parts ownership, and one more posterior, within the temporoparietal cortex, related to the analysis of more global features of BSC, namely self-identification and self-location. BSC conceptualization, traditionally studied in healthy volunteers by manipulating visuotactile stimuli to induce illusory perceptual feelings [see Pyasik et al. (2022[64]) for a review], proved to be useful also in accounting for a wide range of neurological disorders (Ronchi et al., 2018[75]), such as personal neglect (the inability to pay attention to the contralesional hemibody, without concomitant perceptual deficits), somatopraphrenia (the sensation of disowning the contralesional hemibody), autoscopic hallucinations (perceiving an image of oneself in the extrapersonal space). In addition, deficits in BSC have been identified as markers of autism disorder (Mul et al., 2019[54]).

Starting from the relationships between body signals and the self, as underlined by the BSC framework, Riva (2018[69]) made a step forward proposing that not only the bodily self grounds the subjective experience in the online interaction with the world, but the ontogenic process of body memory could be linked to the development of the self. The author identified six main steps, corresponding to as many different representations of the body:

1. ***The sentient body*:** the most basic self-representation is already available at birth. It comprises a topologically-defined invariant structure that, starting during fetal life, integrates interoceptive signals with proprioceptive and vestibular sensations. The output of this representation is the *minimal phenomenal selfhood* (Blanke and Metzinger, 2009[6]) or *protoself* (Damasio, 1999[17]), which is the experience of existing in a sentient body, separated from the external world.
2. ***The spatial body*:** this more sophisticated representation, developed through the first 6 months of life, includes the detection of spatial contingency, integrating afferent sensorimotor information in an egocentric frame of reference. The output of this representation is the *self-location*, that is the ability to localize the body in space (answering the question: “where am I?”).
3. ***The active body:*** it develops in the next six months of life. This map merges afferent sensory information with efferent motor information, thanks to the visuomotor synchrony of the stimuli. Indeed, due to the precise temporal connection between proprioceptive and perceptual information, children become able to perceive visual and proprioceptive sensations as an integrated experience. The active body corresponds to the *core self-proposed* by Damasio (1999[17]). The output of this step is the sense of *agency*, which refers to the recognition of own actions as initiated by the self.
4. ***The personal body*:** around 24 months from birth, kids can incorporate the representation of different body parts into a coherent full-body representation. The output is *full-body ownership*, which is the subjective experience of being the owner of a whole body.
5. ***The objectified body*:** in the next two years a new map is constructed featuring the third-person representation of own body. At this point, the body is represented as an object that can be seen, evaluated, and assessed by others and also by ourselves. This is the precursor of the *autobiographical self* (Damasio, 1999[17]). The outcome of this step is my sense of *mine*, which is the awareness of what belongs to the self, and of the fact that the body is an object of perception to others.
6. ***The social body*:** in the final step of development, the objectified body undergoes an evaluation in light of cultural norms. Therefore, the result is a confrontation between the “actual body” and the “ideal body”, as it is expected to be according to social rules. The outcome of this representation is the *satisfaction/dissatisfaction* brought on by the subjective experience of having a body that is in accordance with or in opposition to social norms (Ideal Me).

According to Riva (2018[69]), these six representations are then incorporated into a coarse supramodal multisensory representation of the body and the space around it, the "body matrix" (Moseley et al., 2012[53]). The body matrix combines different body inputs into a coherent body representation and works in agreement with the predictive coding account (Clark, 2013[14]; Chalk et al., 2018[13]), i.e., it uses a mental model of the body to predict possible incoming inputs and to minimize the amount of surprise [or prediction errors (Apps and Tsakiris, 2014[2])], defined as a mismatch between the individual intentions and actual outcomes of actions accomplished to pursue those intentions. The prediction error minimization makes the interaction between the body matrix and the incoming inputs dynamic (Friston, 2018[25]). On one side, the bottom-up information can actively change the body matrix, to accommodate the unexpected stimuli; on the other side, the body matrix can influence top-down the forthcoming actions aiming at confirming its predictions. An interesting example of the complex balance between stability and malleability of the self during the integration of different bodily signals is provided by Hodossy and Tsakiris (2020[38]). In their study, the congruency of the cardiac signals was altered using a biofeedback paradigm on two hierarchical levels: i.) the low-level congruency between visual feedback and the participant's cardiac signal, and ii.) the high-level congruency between the participant's beliefs about the identity of the cardiac feedback and its actual identity. The dependent measure was the high-frequency heart rate variability (HF-HRV), a marker of phasic vagal cardiac control. The authors found that the HF-HRV was predicted by the congruency on both low-level and high-level hierarchical processing: indeed, lower HF-HRV were detected during incongruent visual feedback compared to the congruent visual feedback; in addition, lower HF-HRV were detected when there was an incongruency between individuals' beliefs and the biofeedback signals. The autonomic responses have been interpreted as *interactions, *namely attempts to minimize the prediction errors, as they unfold following the presence of incongruencies.

At a neural level, precise body matrix localization has not been confirmed yet. Park & Blanke (2019[58]) proposed a cortical system (the x-BSC) supporting the integration of interoceptive and exteroceptive signals and comprising two subsystems, one involved preferentially in self-identification (including the premotor cortex, intraparietal sulcus, and insula), and the second involved preferentially in self-location (including posterior cingulate cortex, intraparietal sulcus, and temporal parietal junction). As underlined by the authors, the two subnetworks overlap within the intraparietal sulcus. Relatedly, a recent metanalysis aiming at unveiling the convergence zone between interoceptive and exteroceptive inputs grounding body awareness identified the bilateral supramarginal gyrus together with a right-lateralized set of areas including precentral, postcentral, and superior temporal gyri (Salvato et al., 2020[76]). 

Recently, several experimental data on healthy individuals and clinical observations have been interpreted in the light of the body matrix framework (Riva and Gaudio, 2018[71]; Brun et al., 2019[10]; Ladda et al., 2020[46]; Crivelli et al., 2021[15]; Riva et al., 2021[72]). For instance, the body matrix is the scaffolding on which to build motor execution and motor imagery training to improve musicians' and dancers' performance, as multimodal processing seems to be the prerequisite for expert performances (Ladda et al., 2020[46]). In addition, the body matrix offered a suitable explanation to account for the complex interaction discovered between exteroceptive (visual stimuli), autonomic (body temperature), and proprioceptive (body position) signals in preserving the sense of body ownership (Crivelli et al., 2021[15]). From a clinical point of view, alterations of the body matrix have been hypothesized as a key factor associated with eating disorders (Riva and Gaudio, 2018[71]; Riva et al., 2021[72]) and pain-related syndromes (Brun et al., 2019[10]; Catley et al., 2019[12]). Riva (2018[69]) has suggested two possible mechanisms through which the bodily experience may be involved in the etiology of different pathologies: i.) a deficit in the capacity to relate bodily signals to their potential positive or negative effects; ii.) a deficit in updating the body matrix with latest information derived from real-time perception. In this line of reasoning, if the damage of the body matrix is, at least partially, responsible for the rising of different psychopathological conditions, then an intervention targeting the body matrix should be employed to re-establish its correct functioning and improve mental health. To this end, Riva (2018[69]) referred to the paradigm of Embodied Medicine (Riva et al., 2017[73], 2019[74]), an approach aiming at using advanced technologies to modify the body matrix to enhance people's well-being. In the next sections, recent experimental pieces of evidence will be illustrated where advanced technologies have been used to affect the body matrix, exploiting two approaches: interoceptive feedback and bodily illusions.

## Altering the Body Matrix through the Manipulation of Interoception

Interoception can be defined as the process by which the nervous system detects, analyzes, and incorporates information coming from the inner body. The purpose of interoception is to create a moment-by-moment map of the interior body on both the conscious and unconscious levels (Khalsa et al., 2018[42]; Quigley et al., 2021[65]). This representation of the internal state of the body is built through the integration of two processes, one ascending from the peripheral nervous system to the central nervous system, aiming at collecting bodily signals to sense the body, and one descending from the central nervous system to the peripheral nervous system aiming at regulating those bodily signals (Schoeller et al., 2022[80]). 

Recently, clinical neuroscience and cognitive neuroscience have paid increasing attention to interoceptive processes as one determinant of physical and psychological clinical conditions (Paulus et al., 2019[59]; Schoeller et al., 2019[79]). Altering the interoceptive feedback, therefore, appears not only useful to better understand the nature of those clinical conditions but also a promising way to build intervention protocols with the ultimate goal of improving health and well-being (Schoeller et al., 2022[80]). These authors suggest three different strategies: i.) artificial sensations, which involve the direct manipulation of interoceptive signals (Riva et al., 2017[73]), ii.) interoceptive illusions, which entail the manipulation of contextual cues to induce a predictable drift in body perception (Iodice et al., 2019[39]), and iii.) emotional augmentation technologies, which combine artificial sensations with personal contextual cues to generate specific moods or emotions (Pezzulo et al., 2018[60]).

As concerns the first strategy, a method to affect the interoceptive feedback is through direct manipulation of interoceptive signals using sound, pressure and/or vibration. This approach has been defined as *sonoception* (Riva et al., 2017[73]). For example, di Lernia and colleagues developed an interoceptive stimulator, used for the stimulation of C-Tactile small unmyelinated fibers (di Lernia et al., 2018[20]). The device provides a slow (at 1-10 cm/s velocities), dynamic (moving along the skin) light-pressure (2.5 mN) to the C-tactile afferents, which are high-sensitive mechanoreceptors known to transmit positive affective aspects of touch (indeed, it is also called *affective touch*) (Ackerley, 2022[1]). In this study, the authors tested the efficacy of the device in enhancing HRV comparing the electrocardiogram signals (ECG) in individuals receiving interoceptive stimulation with those of a control group of participants receiving a static, non-interoceptive pressure. Results confirmed the potential of the device in raising HRV through interoceptive stimulation, opening new avenues of applications in many clinical settings (di Lernia et al., 2018[20]). Relatedly, in a following study, di Lernia and colleagues (2020[21]) applied the interoceptive stimulator to the treatment of chronic pain. Specifically, a group of patients suffering from different forms of chronic pain was randomly assigned to either the experimental condition or the control condition. In the experimental condition, patients received 11 minutes of C-tactile afferents stimulation, whereas, in the control condition, patients received the same amount of static, non-interoceptive pressure. Pain ratings were collected before and after the treatment and revealed a consistent reduction of pain of about 23 % compared to the baseline values only in the experimental group. These promising findings indicate that interoceptive tactile stimulation-based therapies can be useful additional tools for managing pain.

Vibrotactile and thermal stimulation have been recently tested as potential means to improve emotion regulation (Umair et al., 2021[90]). In this study, healthy individuals received vibrotactile and thermal stimulation, delivered by electrodermal actuators, while exposed to a stressful situation. Subjective [i.e. State-Trait Anxiety Questionnaire (Spielberger et al., 1971[88])] and objective measures (HRV) of stress were collected and indicated a suggestive, even though not significant, reduction of stress under the haptic stimulation compared to a control condition. Although this approach has not yet been tested on patients, these preliminary findings seem promising and could pave the way to support emotion regulation in many psychological conditions.

A different approach employing vibrations has been reported in a pilot study by Zhou and coworkers (2021[92]). They tested the potential calming effect of sensing the tactile feedback of one's own heartbeats. During the experiment, a sample of young women received stimulation from a device designed to collect heartbeat from the chest and transform the cardiac signal into tactile vibrations. The task was to stay still, with no additional emotional load. The results indicated a calming effect of the stimulation as evidenced by the participants' HRV, but no changes in their subjective reports of anxiety.

Furthermore, the modulation of the interoceptive signals can be achieved by directly stimulating the brain networks involved in the processing of interoceptive stimuli. One example is provided by the study of Mai and collaborators (2019[49]), who applied repetitive transcranial magnetic stimulation (rTMS) with a continuous theta burst stimulation protocol (cTBS) to two interoceptive network structures, namely the frontotemporal insular network and the somatosensory cortices, with the aim of elucidating the causal role of these structures in emotion processing. The outcome measures were interoceptive accuracy, emotional evaluation of affective pictures and brain correlates of emotional pictures processing. The inhibitory stimulation determined a flattening of the valence judgments, with positive pictures rated as more negative, and negative pictures rated as more positive, especially for the frontotemporal anterior insular stimulation site. This effect was associated with a decreased interoceptive accuracy, indicating that possibly interoceptive prediction errors were prompted by the reduced interoceptive input from bodily signals generated by cTBS in the frontotemporal anterior insular network, which may have led to a conflict between expected and present interoceptive signals. This process, in turn, may be responsible for the reduction of emotional stimuli salience and the related subjective ratings (Seth, 2013[85]; Mai et al., 2019[49]).

The second strategy to modulate interoceptive feedback is modulating the context by delivering exteroceptive cues to generate a predictable outcome. An effective tool to pursue this goal is Virtual Reality (VR). Czub and Kowal (2019[16]) used a virtual avatar to study respiration entrainment. Participants were exposed to a breathing avatar, visualized from the first-person perspective. The avatar initially was breathing at the participants' respiration rate, but after 60 seconds the avatar's breath either speeded up or slowed down, creating a conflict between the participant's respiration rate and the visual feedback. Analyses of the changes in participants' respiration rates evidenced that the disagreements between the visual feedback and respiratory signals have been resolved by means of breath synchronization, thus inducing an entrainment effect. The respiration variation was big enough to reach clinical significance, therefore this approach could be used to support the heart rate variability biofeedback and the diaphragmatic breathing interventions (Czub and Kowal, 2019[16]). Similarly, Solcà and coworkers (2018[86]) applied virtual reality technology with the purpose of targeting interoceptive feedback. In a double-blind experimental study, they had patients suffering from complex regional pain syndrome immersed in a virtual environment depicting their affected limb. Crucially, the virtual limb flashed either synchronously (experimental condition) or asynchronously (control condition) with the patient's real heart rate. The primary outcome measures were the subjective pain ratings, limb strength, and HRV as a physiological marker of pain. The authors found an analgesic effect induced solely by the synchronous feedback, accompanied by a functional improvement of the affected limb and by a modulation of the HRV. These findings confirmed the idea that the painful sensation relies on multiple afferent inputs and can be modulated by providing congruent multisensory information between an observed body and the patient's real body. These effects are thought to be dependent on the mutual connections and partial overlap between the body matrix and central pain representations (the so-called pain matrix, which includes brainstem and thalamic nuclei, primary and secondary somatosensory areas, and insular and anterior cerebellar cortices) (Longo and Haggard, 2012[47]).

The third approach aiming at modulating the interoceptive system is the exploitation of emotional augmentation technologies (Jain et al., 2022[41]; Schoeller et al., 2022[80]) The idea is to link the interoceptive stimulation to the somatic marker of an emotion (Damasio, 1999[17]). This is usually obtained by delivering some audiovisual stimulation in combination with contextual stimuli of personal meaning. One example of this approach is documented in the study by Haar and collaborators (2020[36]). The authors created a device, called Frisson, that generates cold and vibrotactile sensations down individuals' spines in temporal combination with a chill-eliciting auditory stimuli, to enhance the sensation of cold underlying aesthetic chills. The participants underwent the experimental session (with the thermal stimulation) and a control session, whereby the actuator was removed, while experiencing the same audiovisual stimulus. The results indicated that the number and the intensity of the chills increased during the experimental session compared to the control one; in addition, participants reported greater emotional contagion and more pleasure during the session with the device compared to the session without the device. These findings suggest that prosthetic technologies affecting the process of emotion from the bottom-up, effectively impact the subjective experience and could be employed to promote wellbeing. 

## Altering the Body Matrix through the Bodily Illusions

Bodily illusions (BIs) are experiences whereby the perception of one's own body is significantly altered and some body parts, or the whole body, significantly differ from the real body in terms of size, posture, shape, and position (Kilteni et al., 2015[44]). These altered perceptual states can be induced by experimental paradigms that manipulate ad hoc bodily stimuli, usually creating conflicting multisensory information. In some cases, BIs allow embodying fake body parts (i.e. not belonging to the real body) such as in the classical paradigm of the rubber hand illusion (RHI) (Botvinick and Cohen, 1998[9]), when a rubber hand is integrated into the individual body representation after the delivery of a synchronous visuotactile stimulation, over the real and the fake hands. This kind of illusion is made possible by deceiving the sense of embodiment (Arzy et al., 2006[3]; Longo et al., 2008[48]; Kilteni et al., 2012[43]), which corresponds to the sense of having a body and includes three components: body ownership (the sense of being the owner of the body - “this is my body”), self-location (the sense of being located in a specific place - “I am here/there”), and agency (the sense of being the subject who gave the motor command to perform that specific action “I am moving”). In this perspective, it is worth underlying that BIs include both perceptual and motor processes. In a recent review, Dilena and collaborators (2019[23]) summarized the evidence of the effects of BIs on cortical excitability and reported that kinesthetic illusions (induced by vision/tendon vibration) increased corticomotoneuronal excitability, whereas embodying a hand and seeing it moving decreased the excitability of the same neurons.

Advanced technologies such as Virtual Reality have been largely employed to induce bodily illusions with the goal of improving health and well-being (Matamala-Gomez et al., 2021[51]; Turbyne et al., 2021[89]). A key advantage of this tool is the possibility to design the virtual body according to the desired morphological characteristics and the specific goals the clinicians want to achieve. Indeed, it has been demonstrated that VR is capable to affect embodiment not only in healthy individuals, but also in clinical populations (Borrego et al., 2019[8]), opening up a wide range of applications in clinical settings. A first field where VR strongly contributed is pain treatment. Matamala-Gomez and collaborators (2019[50]) studied the effect of embodiment in diverse virtual arms on pain perception in patients suffering from different types of neuropathic pain (namely, complex regional pain syndrome and peripheral nerve injury). The virtual experience included the presentation of a virtual arm co-located as the real one and shown at different transparency levels and sizes. Embodiment measures and pain ratings were collected. The first important finding was that chronic patients can achieve the same level of ownership and agency over the virtual hand as healthy individuals, which is in agreement with the study by Borrego and coworkers on stroke patients (Borrego et al., 2019[8]). Interestingly, touch with a virtual hand reduced pain scores in all patients, but each clinical condition benefited most from a particular type of virtual hand, demonstrating once again the importance of tailoring interventions to patients' specific needs. A different strategy was proposed in a proof-of-concept study by Nighigami and collaborators (2019[55]). In their work, the body illusion was generated by a real-time video footage presented through a head-mounted display. Crucially the video recorded live was altered before its presentation to the participants to induce BI. The setup was designed to reduce lower-back pain in two pilot patients. The camera recorded the patient's body from the rear, showing her back. The software in real time edited the video creating 2 control conditions (normal - no manipulations; reshaped - the shape of the back morphed widening the shoulders and narrowing the waist) and 1 experimental condition (Strong - the shape of the back morphed and merged with an overly of a muscled back). Participants were shown the videos in the three conditions while lifting a weighted basket for a maximum of 60 seconds. Results on pain perception underlined that only one of the two patients reported a decrease in painful sensations in the strong condition compared to the control ones. The authors concluded that the procedure has shown preliminary evidence of potential usefulness in the management of lower back pain, but further studies are needed to clarify which patients' characteristics better predict the efficacy of the treatment. 

In the same line of research, Osumi and coworkers (2019[57]) conducted a study to test the potential of VR-based BIs for restoring phantom limb movement and alleviating phantom limb pain. They had amputees exposed to a virtual experience whereby mirror-reversed computer visual representations of an intact arm-the virtual phantom limb-were shown through a head-mounted display. This caused the illusion of voluntary execution of motions of their phantom limb while intending bimanual actions. Pain intensity was measured through self-report scales, whereas the phantom limb movement was assessed through the bimanual circle line coordination task, in which an incongruency between the task assigned to the real hand (drawing lines) and that assigned to the phantom limb (drawing circles) yields more or fewer deviations in the performance of the real hand depending on the degree of motor representation of the phantom limb (i.e. higher deviations indicate the presence of voluntary phantom limb movements). The authors found a significant restoration of the movement representation and a significant reduction of the pain intensity of the phantom limb after the VR treatment.

A second clinical condition that can be affected by VR-induced BIs is that of eating disorders. The use of BIs in the treatment of eating disorders is VR justified by recent theories (Riva, 2014[68]; Serino et al., 2015[82]; Riva and Dakanalis, 2018[70]; Riva and Gaudio, 2018[71]) according to which these clinical conditions could be related to an impairment in the multisensory body integration, that determine a deficit in updating the body representations (Riva and Gaudio, 2018[71]). In this view, VR can help the patients to update the wrong body representation thanks to the body-swapping strategy (Serino et al., 2016[83], 2019[84]) - a full body illusion where the patient “wears” a virtual body that differs from the real one in size or shape. Recently this approach has been applied to obesity (Scarpina et al., 2019[77]) and anorexia (Porras-Garcia et al., 2020[63]). In the study by Scarpina and co-workers (2019[77]), the aim was to test whether individuals with obesity might successfully experience ownership over a virtual body with a thin belly. Outcome measures were the embodiment ratings and changes in the bodily experience as measured by a body size estimation task. The results indicated that obese patients can experience body ownership over a skinny avatar to the same extent as healthy individuals; in addition, both groups reported changes in the estimation of the body size, in particular the abdomen. The authors underlined that these preliminary findings point to the potential therapeutic use of this approach in patients with obesity.

Similarly, Porras-Garcia and collaborators (2020[63]) applied the body-swapping paradigm to a single patient suffering from anorexia nervosa. She underwent five sessions of VR exposure to a virtual body with an increasing body mass index. Before, and after the treatment ad at the 5-months follow-up researchers collected measures related to fear of gaining weight (FGW), body anxiety, drive for thinness, body image disturbances, body mass index, and body-related attentional bias. The authors reported a reduction of all the symptoms after the intervention, and the maintenance of the improvements at five months, except for the FGW.

Furthermore, full-body illusions have been used also to improve emotion recognition, such as in the study by Seinfeld and collaborators (2018[81]). In their research, a group of male offenders embodied a female victim of domestic abuse from the first-person perspective; the real actions performed by the participants were perfectly mirrored by the virtual actions performed by the avatar. The ability to recognize emotions was tested both before and after the virtual encounter. The results showed that the offenders, after the virtual task, increased their ability to identify fearful female expressions, and decreased their bias towards considering fearful faces as happy. These findings are consistent with the idea that the alterations of the body matrix can affect higher processes such as emotion recognition.

## Conclusions

The aim of this paper was to update current knowledge on body memory by integrating information coming from neuroimaging, cognitive psychology, and clinical research.

Current research is committed to investigating these topics from a theoretical, neurobiological, and clinical point of view. From a theoretical standpoint, the use of different terms (i.e. body image/schema, body self-consciousness, body awareness) to indicate similar concepts around the topic of body representations spread the research efforts toward different and sometimes parallel directions, preventing from building a unifying framework under which reconcile or integrate the different perspectives. The concept of body memory could be a suitable candidate to take on this role. From a neurobiological standpoint, in the last years, there has been an interest in investigating the neural correlates of multisensory integration (i.e. the possible localization of the body matrix), but different studies reported inconsistent findings. From the clinical point of view, an increasing number of studies are being carried out to test the efficacy of advanced technologies for altering the body matrix and improving health, especially in the field of pain management and eating disorders treatment. 

Future research should be done in two complementary but distinct directions. First, more basic research studies should be conducted to validate the body matrix model, testing its predictions possibly with the aid of computational models. Second, even though preliminary evidence testified to the potential use of advanced technologies in the treatment of several pathological conditions, most of the current studies employed very limited samples, with limited reliability. Randomized controlled studies should be encouraged to raise the level of clinical validity, provide clear indications about the best strategies, and identify the categories of patients who could take the most advantage of these treatments.

## Conflict of interest

The authors declare that they have no conflict of interest.

## Figures and Tables

**Figure 1:**
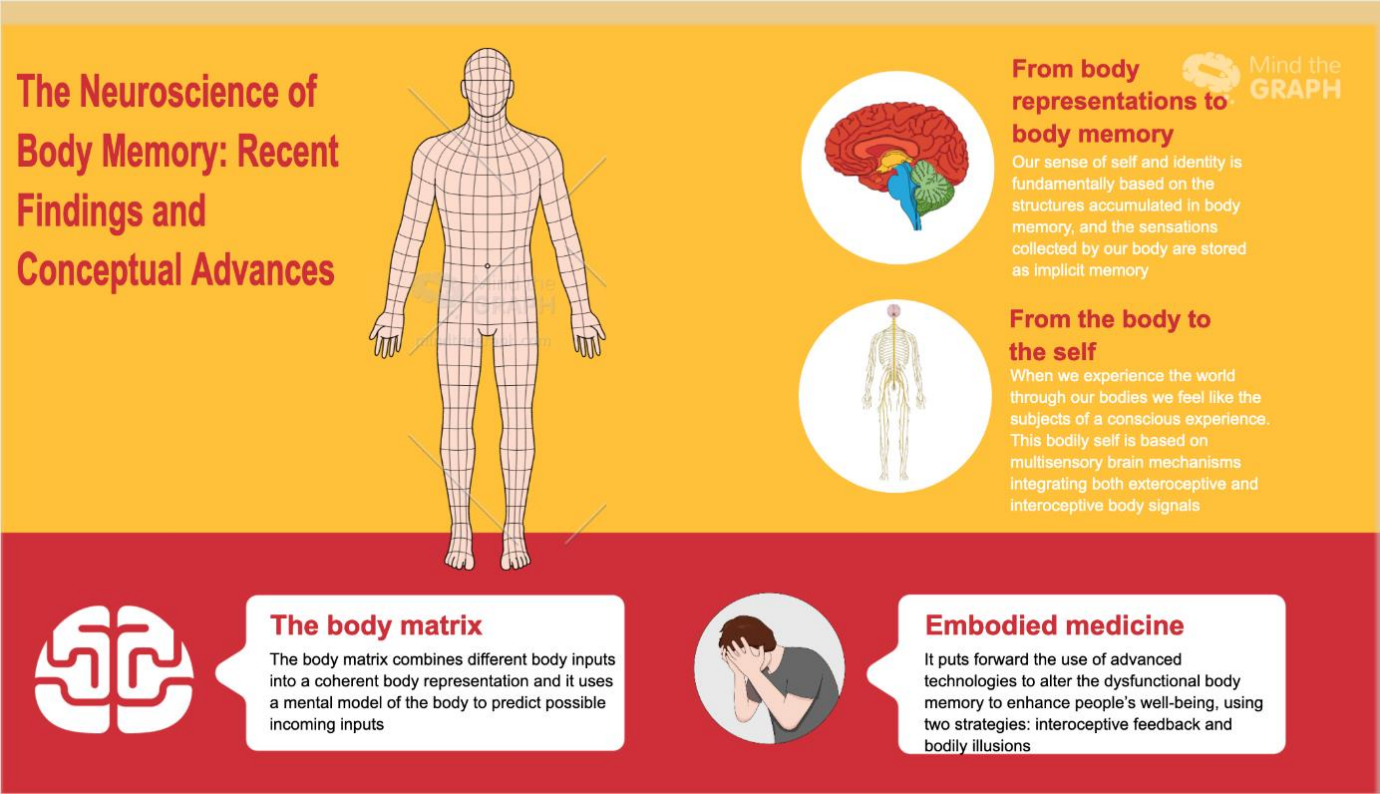
Graphical abstract

## References

[1] Ackerley R (2022). C-tactile (CT) afferents: evidence of their function from microneurography studies in humans. Curr Opin Behav Sci.

[2] Apps MAJ, Tsakiris M (2014). The free-energy self: A predictive coding account of self-recognition. Neurosci Biobehav Rev.

[3] Arzy S, Overney LS, Landis T, Blanke O (2006). Neural mechanisms of embodiment asomatognosia due to premotor cortex damage. Arch Neurol.

[4] Barsalou LW (2008). Grounded cognition. Annu Rev Psychol.

[5] Bergouignan L, Nyberg L, Ehrsson HH (2022). Out-of-body memory encoding causes third-person perspective at recall. J Cogn Psychol.

[6] Blanke O, Metzinger T (2009). Full-body illusions and minimal phenomenal selfhood. Trends Cogn Sci.

[7] Blanke O, Slater M, Serino A (2015). Behavioral, neural, and computational principles of bodily self-consciousness. Neuron.

[8] Borrego A, Latorre J, Alcañiz M, Llorens R (2019). Embodiment and presence in virtual reality after stroke. A comparative study with healthy subjects. Front Neurol.

[9] Botvinick M, Cohen J (1998). Rubber hands ‘feel’ touch that eyes see. Nature.

[10] Brun C, Mercier C, Grieve S, Palmer S, Bailey J, McCabe CS (2019). Sensory disturbances induced by sensorimotor conflicts are higher in complex regional pain syndrome and fibromyalgia compared to arthritis and healthy people, and positively relate to pain intensity. Eur J Pain.

[11] Caggiano P, Cocchini G (2020). The functional body: does body representation reflect functional properties?. Exp Brain Res.

[12] Catley MJ, Moseley LG, Jones MA, Jones MA, Rivett DA (2019). Understanding pain in order to treat patients in pain. Clinical reasoning in musculoskeletal practice.

[13] Chalk M, Marre O, Tkačik G (2018). Toward a unified theory of efficient, predictive, and sparse coding. Proc Natl Acad Sci U S A.

[14] Clark A (2013). Whatever next? Predictive brains, situated agents, and the future of cognitive science. Behav Brain Sci.

[15] Crivelli D, Polimeni E, Crotti D, Bottini G, Salvato G (2021). Bilateral skin temperature drop and warm sensibility decrease following modulation of body part ownership through mirror-box illusion. Cortex.

[16] Czub M, Kowal M (2019). Respiration entrainment in virtual reality by using a breathing avatar. Cyberpsychol Behav Soc Netw.

[17] Damasio A (1999). The feeling of what happens: Body and emotion in the making of consciousness.

[18] de Haan EHF, Dijkerman HC (2020). Somatosensation in the brain: a theoretical re-evaluation and a new model. Trends Cogn Sci.

[19] de Vignemont F (2010). Body schema and body image - Pros and cons. Neuropsychologia.

[20] di Lernia D, Cipresso P, Pedroli E, Riva G (2018). Toward an embodied medicine: A portable device with programmable interoceptive stimulation for heart rate variability enhancement. Sensors (Switzerland).

[21] di Lernia D, Lacerenza M, Ainley V, Riva G (2020). Altered interoceptive perception and the effects of interoceptive analgesia in musculoskeletal, primary, and neuropathic chronic pain conditions. J Pers Med.

[22] Dijkerman HC, de Haan EHF (2007). Somatosensory processing subserving perception and action: Dissociations, interactions, and integration. Behav Brain Sci.

[23] Dilena A, Todd G, Berryman C, Rio E, Stanton TR (2019). What is the effect of bodily illusions on corticomotoneuronal excitability? A systematic review. PLoS One.

[24] Ehrsson HH, Spence C, Passingham RE (2004). That’s my hand! Activity in premotor cortex reflects feeling of ownership of a limb. Science.

[25] Friston K (2018). Does predictive coding have a future?. Nat Neurosci.

[26] Fuchs T, Koch SC, Müller C, Summa M, Fuchs T (2012). Phenomenology of body memory. Body memory, metaphor and movement.

[27] Gadsby S (2019). Body representations and cognitive ontology: Drawing the boundaries of the body image. Conscious Cogn.

[28] Gadsby S (2017). Distorted body representations in anorexia nervosa. Conscious Cogn.

[29] Gallace A, Spence C (2010). The science of interpersonal touch: An overview. Neurosci Biobehav Rev.

[30] Gallagher S (1986). Body image and body schema: A conceptual clarification. J Mind Behav.

[31] Gallagher S (2006). How the body shapes the mind.

[32] Gallese V, Sinigaglia C (2010). The bodily self as power for action. Neuropsychologia.

[33] Galvez-Pol A, Forster B, Calvo-Merino B (2020). Beyond action observation: Neurobehavioral mechanisms of memory for visually perceived bodies and actions. Neurosci Biobehav Rev.

[34] Gaudio S, Quattrocchi CC (2012). Neural basis of a multidimensional model of body image distortion in anorexia nervosa. Neurosci Biobehav Rev.

[35] Gentsch A, Kuehn E (2022). Clinical manifestations of body memories: the impact of past bodily experiences on mental health. Brain Sci.

[36] Haar AJH, Jain A, Schoeller F, Maes P (2020). Augmenting aesthetic chills using a wearable prosthesis improves their downstream effects on reward and social cognition. Sci Rep.

[37] Head H (1920). Studies in neurology Vol. 2.

[38] Hodossy L, Tsakiris M (2020). Wearing your heart on your screen: Investigating congruency-effects in autonomic responses and their role in interoceptive processing during biofeedback. Cognition.

[39] Iodice P, Porciello G, Bufalari I, Barca L, Pezzulo G (2019). An interoceptive illusion of effort induced by false heart-rate feedback. Proc Natl Acad Sci U S A.

[40] Irvine KR, McCarty K, McKenzie KJ, Pollet v T, Cornelissen KK, Tovée MJ (2019). Distorted body image influences body schema in individuals with negative bodily attitudes. Neuropsychologia.

[41] Jain A, Schoeller F, Zhang E, Maes P, Tumuluri R, Sebe N, Pingali G, et al. (2022). Frisson: leveraging metasomatic interactions for generating aesthetic chills. ICMI '22: Proceedings of the 2022 International Conference on Multimodal Interaction, Bengaluru, India, Nov 7-11, 2022.

[42] Khalsa SS, Adolphs R, Cameron OG, Critchley HD, Davenport PW, Feinstein JS (2018). Interoception and mental health: a roadmap. Biol Psychiatry Cogn Neurosci Neuroimaging.

[43] Kilteni K, Groten R, Slater M (2012). The sense of embodiment in virtual reality. Presence: Teleoperators and Virtual Environments.

[44] Kilteni K, Maselli A, Kording KP, Slater M (2015). Over my fake body: Body ownership illusions for studying the multisensory basis of own-body perception. Front Hum Neurosci.

[45] Koch SC, Caldwell C, Fuchs T (2013). On body memory and embodied therapy. Body Mov Dance Psychother.

[46] Ladda AM, Wallwork SB, Lotze M (2020). Multimodal sensory-spatial integration and retrieval of trained motor patterns for body coordination in musicians and dancers. Front Psychol.

[47] Longo MR, Haggard P (2012). Implicit body representations and the conscious body image. Acta Psychol.

[48] Longo MR, Schüür F, Kammers MPM, Tsakiris M, Haggard P (2008). What is embodiment? A psychometric approach. Cognition.

[49] Mai S, Braun J, Probst V, Kammer T, Pollatos O (2019). Changes in emotional processing following interoceptive network stimulation with rTMS. Neuroscience.

[50] Matamala-Gomez M, Diaz Gonzalez AM, Slater M, Sanchez-Vives v M (2019). Decreasing pain ratings in chronic arm pain through changing a virtual body: different strategies for different pain types. J Pain.

[51] Matamala-Gomez M, Maselli A, Malighetti C, Realdon O, Mantovani F, Riva G (2021). Clinical medicine virtual body ownership illusions for mental health: a narrative review. J Clin Med.

[52] Milner D, Goodale M (1995). The visual brain in action.

[53] Moseley GL, Gallace A, Spence C (2012). Bodily illusions in health and disease: Physiological and clinical perspectives and the concept of a cortical “body matrix.”. Neurosci Biobehav Rev.

[54] Mul CL, Cardini F, Stagg SD, Sadeghi Esfahlani S, Kiourtsoglou D, Cardellicchio P (2019). Altered bodily self-consciousness and peripersonal space in autism. Autism.

[55] Nishigami T, Wand BM, Newport R, Ratcliffe N, Themelis K, Moen D (2019). Embodying the illusion of a strong, fit back in people with chronic low back pain. A pilot proof-of-concept study. Musculoskelet Sci Pract.

[56] O’Shaughnessy B (2002). Consciousness and the world.

[57] Osumi M, Inomata K, Inoue Y, Otake Y, Morioka S, Sumitani M (2019). Characteristics of phantom limb pain alleviated with virtual reality rehabilitation. Pain Med.

[58] Park HD, Blanke O (2019). Coupling inner and outer body for self-consciousness. Trends Cogn Sci.

[59] Paulus MP, Feinstein JS, Khalsa SS (2019). An active inference approach to interoceptive psychopathology. Annu Rev Clin Psychol.

[60] Pezzulo G, Iodice P, Barca L, Chausse P, Monceau S, Mermillod M (2018). Increased heart rate after exercise facilitates the processing of fearful but not disgusted faces. Sci Rep.

[61] Pitron V, Alsmith A, de Vignemont F (2018). How do the body schema and the body image interact?. Conscious Cogn.

[62] Pitron V, de Vignemont F (2017). Beyond differences between the body schema and the body image: insights from body hallucinations. Conscious Cogn.

[63] Porras-Garcia B, Serrano-Troncoso E, Carulla-Roig M, Soto-Usera P, Ferrer-Garcia M, Figueras-Puigderrajols N (2020). Virtual reality body exposure therapy for anorexia nervosa. a case report with follow-up results. Front Psychol.

[64] Pyasik M, Ciorli T, Pia L (2022). Full body illusion and cognition: A systematic review of the literature. Neurosci Biobehav Rev.

[65] Quigley KS, Kanoski S, Grill WM, Barrett LF, Tsakiris M (2021). Functions of interoception: from energy regulation to experience of the self. Trends Neurosci.

[66] Raimo S, Boccia M, di Vita A, Cropano M, Guariglia C, Grossi D (2021). The body across adulthood: on the relation between interoception and body representations. Front Neurosci.

[67] Raimo S, di Vita A, Boccia M, Iona T, Cropano M, Gaita M (2021). The body across the lifespan: On the relation between interoceptive sensibility and high-order body representations. Brain Sci.

[68] Riva G (2014). Out of my real body: Cognitive neuroscience meets eating disorders. Front Hum Neurosci.

[69] Riva G (2018). The neuroscience of body memory: From the self through the space to the others. Cortex.

[70] Riva G, Dakanalis A (2018). Altered processing and integration of multisensory bodily representations and signals in eating disorders: A possible path toward the understanding of their underlying causes. Front Hum Neurosci.

[71] Riva G, Gaudio S (2018). Locked to a wrong body: Eating disorders as the outcome of a primary disturbance in multisensory body integration. Conscious Cogn.

[72] Riva G, Serino S, di Lernia D, Pagnini F (2021). Regenerative virtual therapy: the use of multisensory technologies and mindful attention for updating the altered representations of the bodily self. Front Syst Neursci.

[73] Riva G, Serino S, di Lernia D, Pavone EF, Dakanalis A (2017). Embodied medicine: Mens sana in corpore virtuale sano. Front Hum Neurosci.

[74] Riva G, Wiederhold BK, Mantovani F (2019). Neuroscience of virtual reality: from virtual exposure to embodied medicine. Cyberpsychol Behav Soc Netw.

[75] Ronchi R, Park HD, Blanke O (2018). Bodily self-consciousness and its disorders. Handb Clin Neurol.

[76] Salvato G, Richter F, Sedeño L, Bottini G, Paulesu E (2020). Building the bodily self-awareness: Evidence for the convergence between interoceptive and exteroceptive information in a multilevel kernel density analysis study. Hum Brain Mapp.

[77] Scarpina F, Serino S, Keizer A, Chirico A, Scacchi M, Castelnuovo G (2019). The effect of a virtual-reality full-body illusion on body representation in obesity. J Clin Med.

[78] Schenk T, McIntosh RD (2010). Do we have independent visual streams for perception and action?. Cogn Neurosci.

[79] Schoeller F, Haar AJH, Jain A, Maes P (2019). Enhancing human emotions with interoceptive technologies. Phys Life Rev.

[80] Schoeller F, Haar Horowitz A, Jain A, Maes P, Reggente N, Christov-Moore L (2022). Interoceptive technologies for clinical neuroscience. PsyArXiv.

[81] Seinfeld S, Arroyo-Palacios J, Iruretagoyena G, Hortensius R, Zapata LE, Borland D (2018). Offenders become the victim in virtual reality: impact of changing perspective in domestic violence. Sci Rep.

[82] Serino S, Dakanalis A, Gaudio S, Carrà G, Cipresso P, Clerici M (2015). Out of body, out of space: Impaired reference frame processing in eating disorders. Psychiatry Res.

[83] Serino S, Pedroli E, Keizer A, Triberti S, Dakanalis A, Pallavicini F (2016). Virtual reality body swapping: A tool for modifying the allocentric memory of the body. Cyberpsychol Behav Soc Netw.

[84] Serino S, Polli N, Riva G (2019). From avatars to body swapping: The use of virtual reality for assessing and treating body-size distortion in individuals with anorexia. J Clin Psychol.

[85] Seth AK (2013). Interoceptive inference, emotion, and the embodied self. Trends Cogn Sci.

[86] Solcà M, Ronchi R, Bello-Ruiz J, Schmidlin T, Herbelin B, Luthi F (2018). Heartbeat-enhanced immersive virtual reality to treat complex regional pain syndrome. Neurology.

[87] Sorrentino G, Franza M, Zuber C, Blanke O, Serino A, Bassolino M (2021). How ageing shapes body and space representations: A comparison study between healthy young and older adults. Cortex.

[88] Spielberger CD, Gonzalez-Reigosa F, Martinez-Urrutia A, Natalicio LFS, Natalicio DS (1971). The State-Trait Anxiety Inventory. Rev Interam Psicol/Interam J Psychol.

[89] Turbyne C, Goedhart A, de Koning P, Schirmbeck F, Denys D (2021). Systematic review and meta-analysis of virtual reality in mental healthcare: effects of full body illusions on body image disturbance. Front Virtual Real.

[90] Umair M, Sas C, Chalabianloo N, Ersoy C, Ju W, Oehlberg L, Follmer S (2021). Exploring personalized vibrotactile and thermal patterns for affect regulation. DIS '21: Designing Interactive Systems Conference 2021. Virtual Event USA, 28 June 2021- 2 July 2021.

[91] Wilson M (2002). Six views of embodied cognition. Psychon Bull Rev.

[92] Zhou P, Critchley H, Garfinkel S, Gao Y (2021). The conceptualization of emotions across cultures: a model based on interoceptive neuroscience. Neurosci Biobehav Rev.

